# X-ray computed tomography uncovers root–root interactions: quantifying spatial relationships between interacting root systems in three dimensions

**DOI:** 10.3389/fpls.2015.00274

**Published:** 2015-04-29

**Authors:** Alexander M. Paya, Jesse L. Silverberg, Jennifer Padgett, Taryn L. Bauerle

**Affiliations:** ^1^School of Integrative Plant Science, Cornell UniversityIthaca, NY, USA; ^2^Department of Physics, Cornell UniversityIthaca, NY, USA; ^3^School of Electrical and Computer Engineering, Cornell UniversityIthaca, NY, USA

**Keywords:** micro-computed tomography, interspecific interactions, belowground competition, *Picea mariana*, *Populus tremuloides*, 3D root system images

## Abstract

Research in the field of plant biology has recently demonstrated that inter- and intra-specific interactions belowground can dramatically alter root growth. Our aim was to answer questions related to the effect of inter- vs. intra-specific interactions on the growth and utilization of undisturbed space by fine roots within three dimensions (3D) using micro X-ray computed tomography. To achieve this, *Populus tremuloides* (quaking aspen) and *Picea mariana* (black spruce) seedlings were planted into containers as either solitary individuals, or inter-/intra-specific pairs, allowed to grow for 2 months, and 3D metrics developed in order to quantify their use of belowground space. In both aspen and spruce, inter-specific root interactions produced a shift in the vertical distribution of the root system volume, and deepened the average position of root tips when compared to intra-specifically growing seedlings. Inter-specific interactions also increased the minimum distance between root tips belonging to the same root system. There was no effect of belowground interactions on the radial distribution of roots, or the directionality of lateral root growth for either species. In conclusion, we found that significant differences were observed more often when comparing controls (solitary individuals) and paired seedlings (inter- or intra-specific), than when comparing inter- and intra-specifically growing seedlings. This would indicate that competition between neighboring seedlings was more responsible for shifting fine root growth in both species than was neighbor identity. However, significant inter- vs. intra-specific differences were observed, which further emphasizes the importance of biological interactions in competition studies.

## Introduction

Plants sharing a finite amount of space will inevitably interact with each other either above or belowground in the pursuit of essential resources. The outcomes of these interactions can range from positive (facilitation) to negative (competition), and are therefore highly relevant for the development of agricultural and ecological management practices (Grime, 1979; Tilman, 1987). Traditional parameters that quantify the effect of belowground interactions on root growth dynamics include diameter class, spatial/temporal deployment, growth rate, and fine root abundance (Casper and Jackson, 1997; Eissenstat and Yanai, 1997; Eissenstat et al., 2000; Hodge, 2004, 2009; Kembel et al., 2008). While parameters such as these differ across species, accurate *in situ* observations are inherently limited by the opaque and heterogeneous nature of soil matrices, and generally require a destructive harvest of roots (Joslin and Henderson, 1984; Steingrobe et al., 2000), or visualization along a two dimensional (2D) surface (Gross et al., 1993; Majdi, 1996; Eissenstat et al., 2000).

However, recent advances in three dimensional (3D) imaging technology such as ground penetrating radar, laser imaging, nuclear magnetic resonance imaging (MRI), neutron radiography (NT), and X-ray computed tomography (CT) have made the observation of undisturbed root systems possible (Macfall et al., 1991; Butnor et al., 2001; Gregory et al., 2003; Kaestner et al., 2006; Perret et al., 2007; Tracy et al., 2010; Moradi et al., 2011; Mairhofer et al., 2012). Innovations in software such as *Rootviz*, *Root track* (Tracy et al., 2010; Mairhofer et al., 2012), *RootReader3D* (Clark et al., 2011), and *Avizo* (Saoirse et al., 2010), and specific filtering algorithms (Perret et al., 2007) have helped improve 3D image resolution and stream-line the quantification of anatomical parameters such as lateral root length, lateral root number, root-system surface area, and volume of undisturbed root systems. However, accurately isolating roots from root-soil data is complicated by the continuum of water within the root itself, at the root-soil interface, and between soil particles (Lontoc-Roy et al., 2006). As methods for isolating roots improve, steady technological advancements will, and have already increased the scope of viable research questions and objectives. For example, studies have already begun to explore the 3D spatial distribution of fine and coarse roots in forests (Pierret et al., 1999; Butnor et al., 2001), mechanical buckling in plant roots (Silverberg et al., 2012), and water uptake at the root-soil interface (Moradi et al., 2011).

As 3D imaging technologies become more widely available, questions about the occupation and exploration of space by interacting root systems can be better addressed, offering new insights to this important yet problematic component of root growth dynamics. For example, belowground interactions can result in whole root system segregation (reviewed in Schenk et al., 1999), stunted root elongation (Mahall and Callaway, 1996; Falik et al., 2005; Bhatt et al., 2011), and/or over-yielding in response to spatially proximal self-roots (belonging to same plant) and non-self roots (belonging to neighbor) (Gersani et al., 2001; Maina et al., 2002; Falik et al., 2003). An understanding of the mechanisms regulating the growth of roots driven by belowground interactions is still developing, however growing evidence suggests that traditional parameters including root biomass, root surface area, and diameter are insufficient in integrating spatially complex responses.

To our knowledge, the following experiment is the first attempt at observing and quantifying the effect of belowground interactions between two neighboring root systems in 3D. Our research employed micro-CT to capture the spatial distributions of both interacting (inter- vs. intra-specific) and control (solitary) root systems belonging to 2-month-old tree seedlings. 3D models of root system architecture were developed from annotated CT image slices, and traditional belowground parameters such as root length, surface area, volume, and number of root tip were either measured or counted. Moreover, we also developed a series of belowground parameters that take advantage of skeletonized 3D root systems and binary root system data, and evaluate distances between root tips and characterized the distribution of individual root volumes. Broadly, the goal of this work was to investigate the effects of inter- and intra-specific interactions on belowground parameter values, and compare these parameter values with those obtained from solitary individuals.

## Materials and methods

### Plant growth

Acrylic tubes (3.5 mm wall thickness, 64 mm inner diameter, 305 mm length) were covered with fine mesh (0.5 mm) along the base, capped, and secured to provide free drainage. Each tube was filled incrementally with polystyrene beads (1–3 mm), gently tamped throughout the filling process in order to reduce pore size and achieve greater bulk density, and then wrapped in aluminum foil to prevent light penetration. Polystyrene was used in place of peat, sandy loam, sand, or vermiculite based on trail experiments which demonstrated very high water retention in soil or soil like mediums. Additionally, contrast agents such as iodine containing compounds, barium sulfate (BaSO_4_), gold chloride (Au_2_Cl_6_), and cow's milk were used, but sufficient contrast was not achieved.

Black spruce (*Picea mariana*) and quaking aspen (*Populus tremuloides*) were selected as “interacting” plant species based on differences in phylogeny, morphology, and the fact that they co-occur across northern latitudes of North America (DeByle and Winokur, 1985). Seeds from each species were germinated for 5–7 days between two sheets of damp cellulose, and then transplanted into pre-wet hydroponic growth plugs (Rapid Rooster Grow plug™, General hydroponics, Sebastopol, CA) following radicle emergence (1–3 mm). Each acrylic tube received a single plug containing one individual of either spruce or aspen (control), or two plugs, each containing one individual, to simulate inter- or intra-specific interactions. A total of 25 tubes were prepared. There was five of each of the following tubes: solitary aspen, intra-specific aspen, inter-specific aspen/spruce, intra-specific spruce, and solitary spruce. Containers were randomly arranged on a hydroponic flood table modified to re-circulate nutrient solution for top-down irrigation. To prevent competition for light, a sheet of acetate was placed between interacting seedlings.

Plants were grown under greenhouse conditions (17 C° night, 20°C/day; KPL greenhouses, Cornell University, Ithaca NY) with supplemental lighting (12 h/days) for 60 days (April–June, 2012). Irrigation was fed from a 530 liter (140 gallon standard) reservoir to individual micro-spray emitters focused at the base of each plant (90^0^, 0.5 gph, Hydro Flow™, Redmond, WA). Irrigation provided each tree with a nutrient replete hydroponic solution balanced at 150 ppm N (Peter salts, 21-5-20, 382.8 g/530 L; Epsom salts, MgSO_4_ 7H_2_O, 130.64 g/530 L). Hydroponic nutrient solution was maintained at a pH of 5.5–5.8, and electro conductivity of 1.6–2.0 throughout the experiment, and automatically controlled by a programmable timer on a rotating schedule. Half way through the growth phase of the experiment, a pump malfunction resulted in the loss of multiple individuals, which produced an uneven number of replicates for each species.

Irrigation was terminated after 2 months of growth. Plants were allowed to transpire residual water remaining in each container for 2 days prior to imaging in order to reduce imaging artifacts. Plants were then transported to Cornell's imaging facility for CT scanning.

### Micro-CT scans

Whole seedling's root systems were imaged at Cornell University's Micro-CT facility. Due to a pump failure during the growth stage of the experiment, only 13 out of 25 tubes were imaged: 3 × solitary aspen, 1 × intra-specific aspen, 3 × inter-specific aspen/spruce, 3 × intra-specific spruce, and 3 × solitary spruce. Each scan was performed using a GE CT120 micro-CT scanner (GE Healthcare, London, ON, Canada). Initially, 10 bright-field images were acquired with no objects in the scanner, providing a correction for detector non-uniformity. Calibration and correction for signal non-uniformity was determined from measurement within a SB3 (GE Healthcare) water/bone phantom, scanned with the samples. Resulting image datasets were calibrated to the conventional scale of Hounsfiel radiodensity units (HU), defined so that water and air have HU values of 0 and -1000, respectively. Each scan digitally acquired 720 projections at 0.5° intervals over 360° using 80 keV, 32 ma, 32 ms exposure time and 100 μm x-y-z resolution. The obtained projections were used to reconstruct a CT dataset using a convolution back-projection algorithm implemented in 3D, giving a 70 × 70 × 50 mm^3^ volume of image data with 100 μm isotropic voxels.

Using a sequential stacking function (MicroView, GE Healthcare), three sequential image stacks (70 × 70 × 50 mm^3^) were taken from each tube and recompiled into a single 70 × 70 × 150 mm^3^ data set. Using this function, we successfully increased the visible volume (i.e., visible rooting structure) three-fold: from 5 to 15 cm depth (70 × 70 × 150 mm^3^ scan required 1 h of imaging time).

### Destructive harvesting

Following X-ray scanning, plants were destructively harvested. Leaves/needles and petioles were removed from the main stem and scanned using a photo scanner (Epson Expression 10000XL, 2400 dpi, Epson America Inc., Long Beach, CA). Directly following the removal of aboveground tissues, acrylic containers were inverted and tamped to release the polystyrene medium along with roots, which were gently rinsed under a 0.5 mm sieve. Polystyrene beads still attached to roots were removed using forceps. Individual roots were separated manually to prevent overlapping segments, placed on a photo scanner, and scanned. After scanning, above and belowground tissues were placed in separate paper bags, dried at 55 C for 3 days, and then weighed. Scanned images were analyzed for leaf surface area, root surface area, and total root length using *WinRhizo* (Winrhizo 2011, Regent Instruments, Canada). The number of root tips were counted manually using the image analysis toolset, *ImageJ* (National Institute of Health, Bethesda MD). In *ImageJ*, each 2D root system scan was imported as a TIFF, and using the paintbrush tool, root tips were individually and sequentially numbered to ensure that no root tips were overlooked or counted twice.

### Image reconstruction

**Figure 1:**
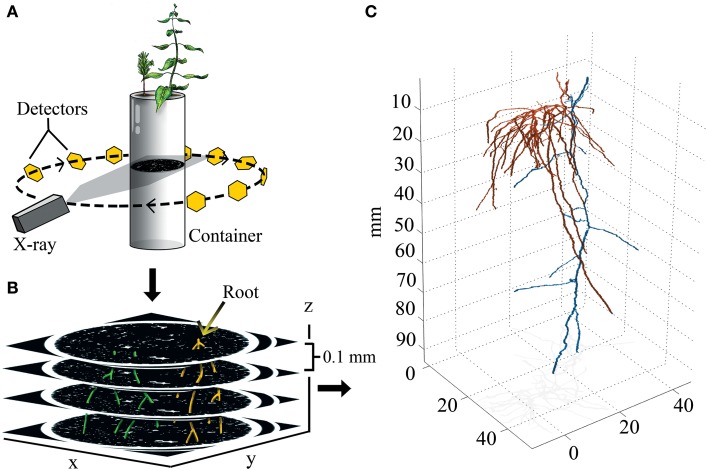
**Experimental design and 3D output. (A)** Drawing representing the principles of X-ray CT. X-rays are aimed at a container, and the signal attenuation of the X-ray beam is captured by a ring of detectors that integrate signal information into image slices made of isotropic voxels (0.05 × 0.05 × 0.1 mm). **(B)** Identifiable roots are color coded, and then reconstructed in 3D. **(C)** 3D reconstruction of paired aspen (*Populus tremuloides*, orange) and spruce (*Picea abies*, blue) root systems.

Projections were exported from the GE CT120 micro-CT scanner as VFF format (Sun TAAC Graphic File) and converted to DICOM format using MicroView's DICOM transfer tool. Image stacks were then imported one at a time into *ImageJ* using the *import, image sequence* function. Once the main taproot was found for each seedling, roots originating from a single individual seedling were given an arbitrary color code, and the entire cross sectional area was traced by hand for each root through each CT image slice (70 × 70 × 0.1 mm) (Figure 1). This ensured that root diameter, surface area, and volume could be measured in the 3D model/reconstruction, and that root systems belonging to different individuals could be easily differentiated. Once a root came in contact with the container wall, tracing ceased because, (1) the roots were indiscernible from the container, and (2) the roots behaved atypically and tracked the container wall. Color-coded image stacks were then exported as an RGB TIFF stack, and opened in MATLAB® 2012b for three dimensional reconstruction and spatial quantification (The MathWorks Inc., Natick MA). In order to 3D render each root system, color codes were identified and isolated, which allowed for the subtraction of non-colored voxels; annotated circles representing root cross sections within each x-y plane were then stacked across the z-plane. This process effectively rendered each root system in 3D with little or no constraints on actual root system dimensions. In MATLAB each root cross section was also converted to a 3D binary matrix in order to measure spatially explicit parameters. Entries of these matrices were either 0 or 1, depending on whether that voxel was occupied by the root.

### Morphological and anatomical analyses

Root surface area was determined from the 3D data sets by sequentially analyzing each x-y cross-section with MATLAB's *bwtraceboundary* function. This identified the coordinates of the root perimeter from which we calculated the circumference of all roots passing through the plane. The circumference was multiplied by the cross-sectional thickness (100 μm) to estimate root surface area per image slice. This was performed for all cross-sectional images and the results summed to calculate root system surface area. Root system volume was calculated by summing the total number of occupied voxels and multiplying by the volume per voxel, 10^−3^ mm^3^/voxel.

Root tips were located by scanning through each cross-section and identifying terminal voxels. This generated a list of coordinates that were centered on root tips (Figure 1). With this information we were able to determine the depth-wise distribution along the z-axis (complete 3D information). To quantify the radial distribution of root tips (mm^2^), root tip coordinate data was projected into the x-y plane. An ellipse whose circumference and orientation represents the occupied x-y area of the root system was then projected over the root tip coordinates. Multiplying π by both the major and minor axes of the ellipse provided the radial distribution of each root system. The ratio of the ellipse's major to minor axis is then a metric that defines how radially symmetric the root distribution is. In particular, if the ratio is 1, then the distribution is circularly symmetric. Values higher than 1 indicate the amount of asymmetric root growth in the plane that passes vertically through the ellipse's major axis.

### Statistics

In order to validate our 3D rendering protocol, we used simple linear regression to compare destructively harvested (2D) and 3D reconstructed root system data. Surface area and the number of root tips were chosen for regression because both were measurable in 2D and 3D, and could therefore be used to validate our manual tracing procedure of fine root cross sections through CT image slices. The intercept (a) in the simple linear regression model (y = a + bx + error) was constrained to be equal to 0, and thus was not estimated (see **Figure 3**). Differences in mean ranks between solitary, intra- and inter-specifically growing plants were analyzed using the Kruskal-Wallis test on the following parameters: biomass, 2D/3D root length, 2D/3D surface area, specific root area (SRA), specific root length (SRL), manual and 3D root tip count, 3D root volume, root–root distance, major/minor axes, radial distribution, and rooting depth weighted by volume (*P* = 0.05, H_o_ = mean ranks are equal). Where the null was rejected, *post-hoc* analyses were performed using the Wilcoxon each-pair test (non-parametric multiple comparisons, *P* = 0.01667). It is important to note that the distribution of minimum Euclidean distances between root tips did not distribute normally, nor did the data behave normally post-transformations (e.g., log x, e^x^, or x^−1^). Instead, we used a two-parameter model for the probability distribution function that fit both species data where the ratio of the sum of squares of regression (SS_reg_) to the total sum of squares (SS_tot_) was between 0.75 and 0.99. This model was a good predictor of the fraction of root tips *f(x) dx* separated by a minimum distance between *x* and *x* + *dx* for control, inter-, and intra-specifically growing seedlings, where the *pdf* is given by:

(1) $$f(x)=c_{1}xe^{-x/c_{2}}.$$

Non-linear regression models were fitted using Sigma Plot 11 (Systat Software, San Jose, CA). All other statistics were done using JMP 10.0 (SAS Institute Inc., Cary, NC).

## Results

### Destructive analyses

**Figure 2:**
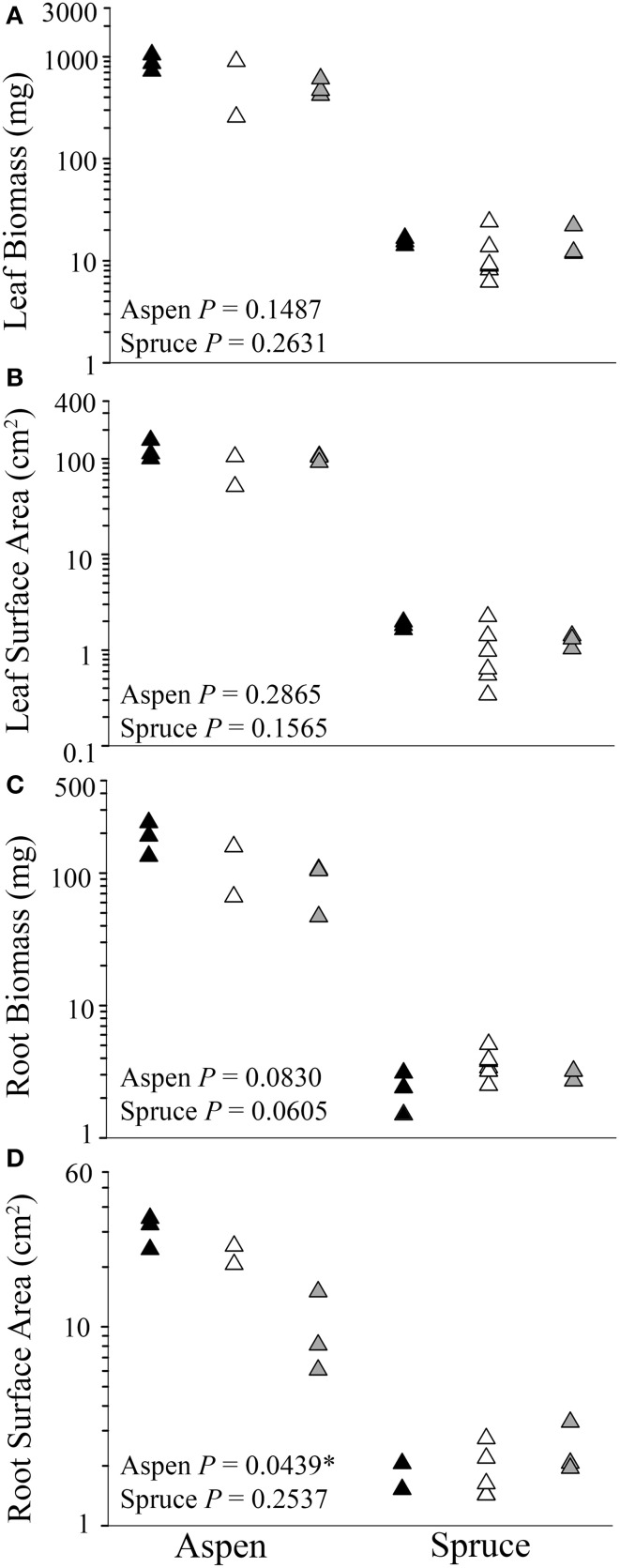
**Differences between control (solitary individuals: black) and paired seedlings (intra-specific: white, or inter-specific: gray) in aboveground (A,B) and belowground (C, D) biomass and surface area of aspen (left) (*Populus tremuloides*) and spruce (right) (*Picea mariana*) post-destructive harvest**. Each point (triangle) represents data from a single individual displayed on a log scale (y-axis); ^*^ denotes significant differences among control, intra-, and inter-specific seedlings (Kruskal-Wallis test, *P* ≤ 0.05).

Analyses of each harvested seedling showed that belowground interactions had no significant effect on the aboveground growth of aspen or spruce (*P* = 0.1487, Figures 2A,B). Belowground interactions had no significant effect on either species' root biomass (*P* = 0.0606, Figure 2C). Belowground interactions had a measurable effect on aspen's root surface area, but not spruce (*P* = 0.0439, Figure 2D). On average, inter-specific aspen root systems were reduced to 31% of the control samples surface area. Lastly, belowground interactions had a significant effect on fine root lengths in aspen (*P* = 0.0439), but not spruce (*P* = 0.2120).

Two additional belowground parameters, SRL (cm mg^−1^) and SRA (cm^2^ mg^−1^), were also calculated for both species. SRL and SRA depict the cost of root construction, and can be highly informative in establishing whether a treatment had an effect on root morphology. Control, inter-, intra-specifically growing spruce seedlings differed in terms of SRA (*P* = 0.0389), but similar differences were not observed in aspen (*P* = 0.0783, Table 1). Control, inter-, and intra-specific interactions also had a significant effect on the SRL of spruce seedlings (*P* = 0.0134), but not aspen (*P* = 0.2082).

**Table 1:** **Belowground parameters of destructively harvested aspen (*Populus tremuloides*) and spruce (*Picea mariana*) seedlings grown under three experimental conditions: control, intra-specific, and inter-specific**.

		***n***	**Root biomass (mg)**	**Root surface area (cm^2^)**	**Number of root tips**	**Root length (cm)**	**SRA (cm mg^−1^)**	**SRL (cm^2^ mg^−1^)**
Aspen	Control	3	192 (241, 135)	32.7 (35.2, 24.5)	266 (315, 181)	693 (724, 528)	0.170 (0.180, 0.150)	3.79 (5.16, 2.19)
	Intra	2	113 (159, 66.4)	22.3 (23.9, 20.6)	173 (195, 152)	424 (443, 405)	0.230 (0.310, 0.150)	4.62 (6.68, 2.55)
	Inter	3	77.3 (106, 47.7)	8.11 (15.0, 6.07)	114 (142, 50)	168 (241, 107)	0.130 (0.140, 0.080)	2.26 (2.27, 1.61)
Spruce	Control	3	2.40 (3.10, 1.50)	1.52 (2.06, 0.529)	12.0 (15.0, 7.00)	19.3 (25.5, 14.2)	0.640 (0.660, 0.350)	8.23 (9.47, 8.06)
	Intra	6	3.60 (5.10, 2.50)	1.53 (2.75, 0.742)	15.0 (28.0, 6.0)	18.1 (28.5, 9.87)	0.415 (0.700, 0.300)	4.81 (7.32, 3.72)
	Inter	3	2.95 (3.28, 2.70)	2.07 (3.32, 1.95)	12.0 (22.0, 8.00)	27.7 (35.0, 22.2)	0.770 (1.04, 0.720)	10.2 (10.9, 8.21)

*Values listed are the median (maximum, minimum)*.

### Validation of 3D reconstruction

**Figure 3:**
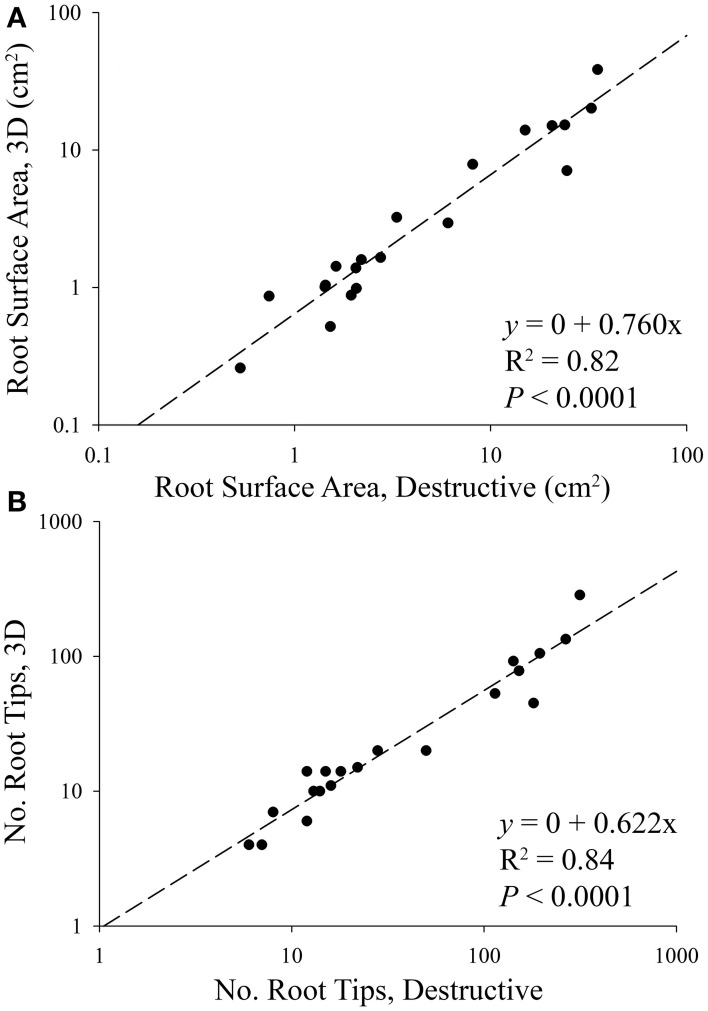
**Linear regressions between destructive and 3D (A) root surface area and (B) the number of root tips of aspen (*Populus tremuloides*) and spruce (*Picea mariana*) seedlings**. The regressions were performed using combined species data. The slope of each regression indicates that 76% of root surface area, and 62% of root tips were successfully rendered in 3D, i.e., 24–38% of root systems were lost during the 2D annotation and 3D reconstruction phase of the experiment. Based on differences in aspen and spruce root system size, data points are displayed on a log scale only to better visualize smaller root systems.

**Figure 4:**
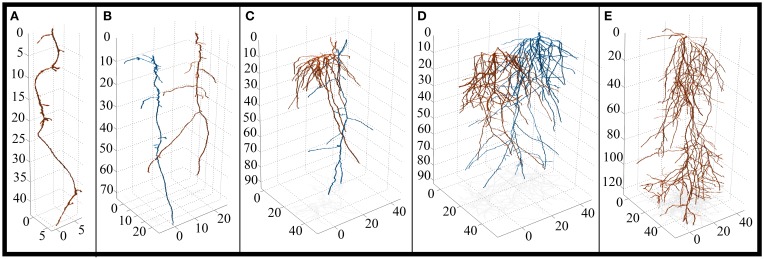
**3D reconstruction of solitary and paired root systems (axes in 0.1 mm increments). (A)** Control spruce (*Picea mariana*) (*n* = 3 containers, 3 individuals). **(B)** Intra-specific spruce (*n* = 3 containers, 6 individuals). **(C)** Inter-specific aspen (*Populus tremuloides*) and spruce root systems (*n* = 3 containers, 3 individuals each species). Aspen is highlighted in orange, spruce is highlighted in blue. **(D)** Intra-specific aspen (*n* = 1 container, 2 individuals). **(E)** Control aspen (*n* = 3 containers, 3 individuals). Note the differences in x and y-axes. For a complete list of 3D reconstructions, refer to Figure S1.

In order to validate our 3D reconstruction protocol, comparisons were made between destructive (i.e., 2D) and 3D root parameters. Surface area and the number of root tips were two parameters measured in both 2D and 3D, and were therefore chosen to validate the 3D reconstruction by way of simple linear regression. We found that 62% of the total number of root tips, and 76% of the total surface area were successfully captured during 3D image reconstruction (Figure 3). Examples of 3D root systems form each species combination are presented in Figure 4, as well as Figure S1.

### 3D utilization of space

The occupation of 3D space by individual root tips, and whole root systems was quantified via a set of five metrics: radial distribution of root tips, directionality (major/minor axes of radial distribution ellipse), minimum root–root tip distance, root system volume as a function of depth, and the vertical position of root tips. The radial distribution of root tips measured the radial expanse of a root system (mm^2^), i.e., the area of an ellipse that encompassed the x/y distribution of all root tips projected along the z-axis. The radial distribution of aspen roots ranged from 248 to 501, 500 to 522, and 212 to 325 mm^2^ in the control, intra-specific, and inter-specific seedlings, respectively (Table 2). For spruce, the control, intra-specific, and inter-specific seedlings ranged from 16 to 43, 9 to 112, and 36 to 314 mm^2^, respectively (Table 2). We found no significant effect of belowground interactions on the radial distribution of roots.

**Table 2:** **Measurements made in 3D of aspen (*Populus tremuloides*) and spruce (*Picea mariana*) seedlings grown under three experimental conditions: control, intra-specific, and inter-specific**.

		***n***	**Root volume (mm^3^)**	**Root surface area (cm^2^)**	**Number of root tips**	**Major/Minor radii**	**Radial distribution (mm^2^)**
Aspen	Control	3	230 (424, 84.0)	20.1 (38.4, 7.06)	134 (285, 45.0)	1.16 (1.38, 1.09)	484 (501, 248)
	Intra	2	159 (161, 157)	15.1 (15.2, 15.0)	91.5 (105, 78.0)	1.09 (1.10, 1.07)	511 (522, 499)
	Inter	3	88.5 (158, 33.4)	7.87 (13.9, 2.94)	53.0 (92.0, 20.0)	1.38 (1.57, 1.28)	263 (325, 212)
Spruce	Control	3	4.20 (11.7, 2.16)	0.519 (1.39, 0.259)	14.0 (14.0, 4.0)	1.54 (10.1, 1.19)	25.4 (43.4, 16.4)
	Intra	6	10.5 (21.0, 4.00)	1.23 (1.65, 0.863)	10.5 (15.5, 8.50)	3.37 (6.31, 1.78)	40.6 (112, 9.16)
	Inter	3	11.3 (37.7, 7.77)	0.984 (3.23, 0.878)	7.00 (15.0, 6.00)	2.70 (5.55, 1.46)	47.4 (313, 36.3)

*Values listed are the median (maximum, minimum)*.

The directional growth of roots was measured by dividing the major (transverse) by the minor (conjugate) axes of an ellipse that encompassed the radial distribution of each root system. Using this approach, we could determine whether a root system was concentric (major/minor = 1), or skewed/directional (e.g., major/minor >> 1). As was the case with the radial distribution of root tips, belowground interactions had no significant effect on the ratio of major/minor axes for either species. However, notable species trends were observed. The major/minor axes of spruce root systems ranged from 1 to 10, with a mean of 4 across solitary and paired seedlings, indicating that spruce roots systems were relatively planar (Table 2). The major/minor axes of aspen root systems ranged from 1.1 to 1.6, with a mean of 1.2 across control, inter- and intra-specific seedlings, indicating that aspen root systems were evenly distributed in all directions relative to each root system's center of mass.

**Figure 5:**
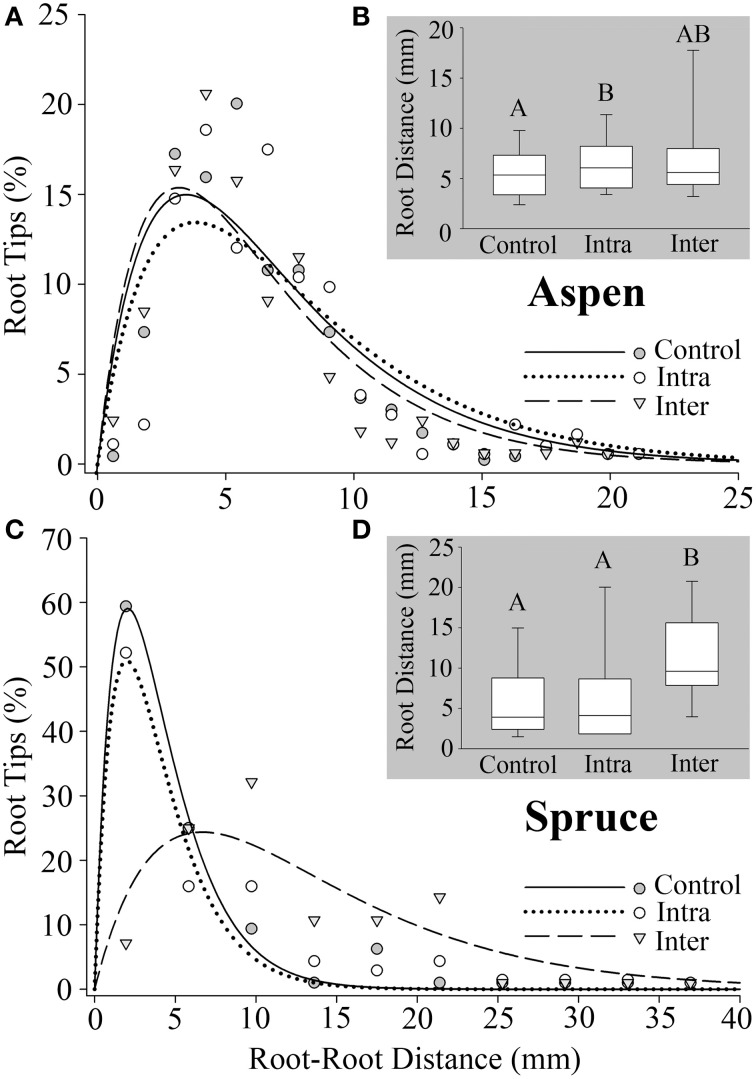
**Minimum distance between a root tip (x_1_, y_1_, z_1_) and the closest tip (x_2_, y_2_, z_2_) calculated for all roots. (A)** Aspen (*Populus tremuloides*) control (SS_reg_/SS_tot_ = 0.86), intra-specific (SS_reg_/SS_tot_ = 0.77), and inter-specific (SS_reg_/SS_tot_ = 0.86). **(C)** Spruce (*Picea mariana*) control (SS_reg_/SS_tot_ = 0.99), intra-specific (SS_reg_/SS_tot_ = 0.91), and inter-specific (SS_reg_/SS_tot_ = 0.75). **(B,D)** Box plots representing root–root tip distances for aspen and spruce, respectively. Each box displays the median value (straight line) and upper/lower quartiles; bars represent 10th/90th percentile. Two median values that do not share a common letter **(A,B)** differ significantly (Wilcoxon multiple comparisons, *P* ≤ 0.01667). Note the differences in axes.

The third metric, minimum root–root tip distance, measured the minimum distance between a root tip (x_1_, y_1_, z_1_) and the nearest neighboring root tip (x_2_, y_2_, z_2_) for every terminal point in a root system. Belowground interactions had a significant effect on the minimum distance between root tips in both aspen (*P* = 0.0114) and spruce (*P* = 0.0002). *Post-hoc* analyses indicated that aspen roots grown intra-specifically (6.8 ± 0.28 mm) had wider distances between root tips compared to the controls (5.9 ± 0.13 mm) (*P* = 0.0025, Figure 5B). As for spruce, *post-hoc* analyses indicated that the minimum distances between root tips in controls (6.0 ± 0.78 mm) were significantly less than inter-specific seedlings (11 ± 1.0 mm, *P* < 0.0001, Figure 5D). The minimum distances between root tips in intra-specific seedlings (7.8 ± 0.92 mm) were also significantly less than inter-specific seedlings (*P* = 0.0007, Figure 5D).

**Figure 6:**
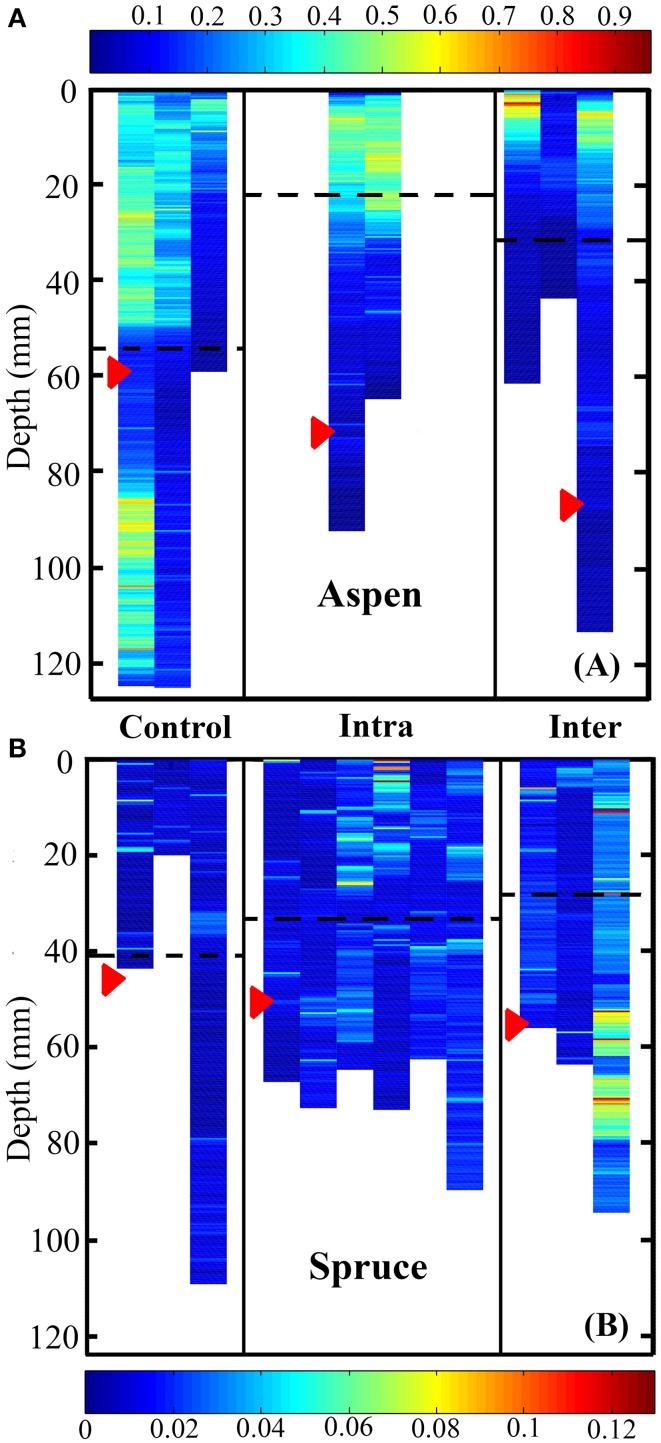
**Heat map representing root system volume as a function of depth for aspen (A, *Populus tremuloides*) and spruce seedlings (B, *Picea mariana*)**. Control, intra-, and inter-specific interactions were significant predictors of rooting depth weighted by volume for both aspen and spruce (Kruskal-Wallis test, *P* < 0.0001). *Post-hoc* analyses indicated that in aspen, control, inter-, and intra-specifically growing seedlings were all significantly different in terms of their rooting depth weighted by volume (*P* < 0.0001). In spruce, *post-hoc* analyses indicated that inter-specific vs. control plants, as well as control vs. inter-specific plants were significantly different in terms of rooting depth weighted by volume (*P* < 0.0001). Units are in mm^3^. Each striated column represents the full root volume of a single seedling. Red arrows indicate the mean depth of root tips, while striped black lines indicate the mean depth of root system volume.

We also quantified differences between control, inter-, and intra-specifically growing plants in terms of their vertical placement of root system volume (Figures 6A,B). For both species, control, inter-, and intra-specific interactions were significant predictors of the depth of roots weighted by volume (Kruskal-Wallis test, *P* < 0.0001). In aspen, *post-hoc* analyses indicated that the largest difference was observed between control (deep) and intra-specific (shallow) root systems (*P* < 0.0001). The second largest difference in rooting depth was observed between inter- and intra-specifically growing seedlings (*P* < 0.0001). The smallest difference was observed between inter-specific and control root systems (*P* < 0.0001). In spruce, *post-hoc* analyses indicated that the largest difference was observed between inter- and intra-specifically growing root systems (*P* < 0.0001). Inter-specific and control root systems also occupied significantly different rooting depths (*P* < 0.0001), while no significant difference was observed between intra-specific and control root systems (*P* = 0.0864). The mean rooting depth for control, intra-specific, and inter-specifically growing root systems for aspen was 56.2 ± 0.6, 23.6 ± 0.6, and 32.7 ± 0.7 mm, respectively, whereas spruce was 41.0 ± 2.4, 34.4 ± 1.2, and 28.6 ± 2.31 mm, respectively.

The vertical distribution of aspen root tips differed significantly between control, intra-, and inter-specifically growing seedlings (*P* < 0.0001). Solitary aspen tended to distribute their root tips evenly across vertical space, and occupied a mean depth of 58.6 mm ± 1.43 mm. Intra-specifically growing aspen root tips were predominately located between 300 and 1200 mm, and occupied a mean depth of 72.5 ± 3.01 mm. Inter-specifically growing aspen root tips were concentrated between 400 and 1000 mm, and occupied a mean depth of 85.6 ± 2.41 mm. *Post-hoc* analyses indicated that inter-specific root tips were located significantly deeper than intra-specific root tips (*P* < 0.0001), as well as control root tips (*P* < 0.0001). Intra-specific root tips were also located significantly deeper than control root tips (*P* = 0.0017). The mean depths of spruce root tips did not differ between control, intra- and inter-specific seedlings, which were located at 45.2 ± 6.56, 50.4 ± 2.86, and 58.2 ± 6.10 mm, respectively.

## Discussion

### The effect of belowground interactions on root growth

In this experiment, each seedling was grown under full nutrient conditions without competition for light to minimize any variation that could be attributed to aboveground resource competition between solitary and paired individuals. The results of this study suggest that belowground interactions between neighboring root systems had measurable effects on root system architecture and 3D distribution of fine roots in the two species. For example in aspen, inter-specific interactions reduced belowground surface area, root length, and SRL when compared to solitary aspen seedlings, which suggests that inter-specific interactions negatively affect aspen root system growth (Table 1). In contrast, inter-specifically growing spruce seedlings, when compared to solitary spruce seedlings, increased root surface area, root length, SRA, and SRL (Table 1). This trend was not observed when comparing solitary and intra-specifically growing spruce seedlings, which suggests that spruce may over-yield under inter-specific growing conditions (Brassard et al., 2011).

We found that belowground competition shifted the 3D distribution of aspen roots. One example was root–root tip distances; the minimum distance between neighboring root tips under inter-specific conditions (7.5 mm) was greater than controls (5.9 mm, Figure 5). While a 1.6 mm difference in spacing may seem small, phosphorus concentrations increase exponentially over a distance of 1 mm from a root's surface (Hendriks et al., 1981). Therefore, relatively small adjustments in the spacing of root tips could result in distinct levels of competition for nutrients in soil (Hodge, 2009). The observed variation in root–root spacing may also result from a lower average number of root tips per inter-specific root system (102 tips) compared to intra-specific (173 tips) or controls (254 tips). Given a constrained volume, a reduction in the number of root tips could result in greater distances between them.

We also observed that the vertical placement of aspen's roots differed between the control, intra-, and inter-specific seedlings (Figures 6A,B). This observation suggests that aspen can respond within 2 months of germination, and with high plasticity, to the presence of a neighboring root system by shifting the vertical placement of its roots. These changes in fine root vertical distribution may result in distinctly different rooting depths between aspen and inter-specific neighbors, thus reducing belowground competition (Schenk et al., 1999; Schenk, 2006). Alternatively, shifts in aspen's vertical root distribution may result in root system overlap with neighboring plants establishing direct competition. Regardless of whether aspen's strategy is to outcompete, avoid competition, or some combination of both in response to a neighboring plant, we found that the presence of both inter- and intra-specific neighbors is sufficient to alter the vertical growth of aspen roots. Care must be taken in the interpretation of these results, however, as specific changes in the occupancy of space by inter- and intra-specifically growing root systems may result from their being planted side-by-side and not in the center of each container.

Belowground interactions also shifted the vertical positioning of aspen root tips, and to a lesser extent in spruce. In both solitary and paired aspen seedlings, the whole root system's mean rooting depth was shallower than the mean depth of root tips alone. Specifically, inter-specific aspen exhibited the greatest difference between mean root system depth and mean root tip depth (Figure 6). Under inter-specific conditions, the mean depth of root tips (85.6 mm) was markedly deeper than the mean depth of the whole root system (32.7 mm). This observable difference between the mean depth of an entire root system, and the mean depth of it's root tips is noteworthy, mainly because not all roots within a root system are functionally equivalent (Pregitzer et al., 1997). Especially in woody plants, a large proportion of a root system is in the form of long-lived, woody roots that anchor the plant and support essential transport functions, as opposed to the most distal part of the root system, the root tips, which are highly metabolically active and demonstrate the highest rates of nutrient and water uptake among all root classes (Pregitzer et al., 1997; Volder et al., 2005). By reducing overlap among “transport” roots and root tips, a root system can occupy an exclusive volume of soil space while simultaneously foraging for resources, and minimizing competition with itself. Therefore, when quantifying root growth dynamics in 3D volumes, either in response to itself or non-self interactions belowground, special attention should be paid to the dynamic growth and placement of root tips independently of whole root systems.

The root system architecture of spruce was dominated by a main taproot with relatively few lateral roots (Figure 4), which resulted in very few significant differences between treatments. However, there were some notable trends worth discussing. SRA and SRL tended to be higher under inter-specific conditions compared to intra-specific conditions, as well as solitary individuals, indicating a lower cost of construction for spruce roots under inter-specific conditions. Also, inter-specific spruce roots tended to grow deeper, place root tips deeper, and root tips were spaced farther apart when compared to solitary plants—a response that was similar to aspen.

### Modeling root–root interactions

The use of mathematical models to describe biological phenomena is inherently complicated by the nature of organismal responses to heterogeneously distributed biotic and abiotic cues (Hodge, 2009), though in the context of root systems, both mechanical (Moulia, 2013), and fractal analysis (Tatsumi et al., 1989; Fitter and Stickland, 1992; Ozier-Lafontaine et al., 1999) have been applied with some success. Belowground, root system responses can manifest as a proliferation of roots into a nutrient rich patch (Robinson et al., 1999), altered root morphology (Bolte and Villanueva, 2006), or shifts in the direction of growth (Falik et al., 2005). Accurately modeling this type of non-random growth response is possible (e.g., Godin, 2000), but requires data that is highly resolved, both spatially and temporally. In this study, we demonstrate high spatial resolution for a single point in time, which limits our ability to quantify dynamic growth processes. Nevertheless, 3D structural information, such as that captured by the micro-CT techniques used here, provides insights otherwise inaccessible with 2D destructive imaging. We support that these findings can be used to verify or refute predictions of derived equations that incorporate the interactions between plants.

When modeling root–root distances, we discovered that a phenomenological exponential “growth and decay” model fit well for both species (SS_reg_/SS_tot_ = 0.75–0.99). This model was chosen from a number of mathematical models that were developed to accurately describe the distribution of root–root distances. Alternative models that were generated but not included in this study tended to fit the data somewhat better, but were species specific. This 2-parameter model was adopted because it accurately described the distribution of data for both species across all belowground conditions, whereas other similarly simple models could not. When developing and applying such models, it is important to keep in mind not only the model's fit, but the scope of the experimental question, the complexity of the model, its predictive value, and whether it can be applied broadly across species.

Also, based on equal amounts of nutrient, water, and light supplied to each container housing either one or two individual seedlings, and the equidistant orientation of paired individuals (intra- and inter-), we assumed that belowground interactions (intra- vs. inter-) would have the same effect on each paired seedling. There is no way for us to know with certainty that individuals were experiencing a treatment effect, or simply resource competition, which would result in similar belowground outcomes. However, as previously mentioned, nutrient levels were maintained at an EC of 1.6–2.0 throughout the experiment, reducing the likelihood of resource competition throughout the experiment. Future experiments should aim to parse out the different effects of nutrient competition and non-resource interactions.

### Comparing methods and constraints

Previous attempts to image undisturbed root systems have been met with mixed success. The current benchmark for successfully rendering roots in a 3D data set is set at roughly 90%. For example, Gregory et al. (2003) captured 90% of 7-day-old wheat roots that did not exceed 10 cm in total length. Kaestner et al. (2006) reported that they could successfully capture 90% of *Alnus incana* (alder) roots larger than 0.18 mm. However, alder roots had to first be removed from their growing medium and packed into quartz sand prior to imaging. In another experiment, Perret et al. (2007) captured around 87% of the total root segments, and 78% of the total root length in 21-day-old chickpea.

In our experiment using *P. mariana* (spruce) and *P. tremuloides* (aspen), we successfully rendered between 62 and 76% of the actual root system architecture (Figure 3). We believe that roughly 30% of the root systems were lost in the manual root tracing/annotation phase of the methodology because of the criteria we followed for each annotation. Specifically, roots that contacted the container wall were excluded on the basis that these roots behave uncharacteristically, i.e., container tracking and circling. We predicted that based on aspen's larger root system size, a larger proportion of aspen roots would have been lost in the reconstruction phase when compared to spruce. However, data from both species were included in our general linear model (Figure 3). We found that both species realized a similar percentage loss of roots, which suggests that any bias introduced as a result of the annotation criteria was similar for both species.

Limitations of our methodology include (1) the use of synthetic growth medium, (2) manual identification of roots in the annotation process, and (3) the relatively small instrument aperture. Regretfully, we could not heed the call of Gregory and Hinsinger (1999), who argued that future advancements in research involving micro-CT and plant roots must focus on using natural soils in place of sand or hydroponics. Distinguishing between water within roots, and water in the medium is an often-reported limitation—one we experienced early on during method development. We attempted to circumvent this issue by growing plants in hydrophobic, synthetic “sand.” This way, the amount of water remaining in the container during imaging would be minimized, facilitating root identification. While this worked well for us, we cannot conclude that either species' growth was unaffected by this growth method. Though residual water was minimized, there were still trace amounts that disrupted several automated root-tracking algorithms that were developed during this experiment. Thus, the data required manually tracing each root through +1400 CT image slices, which required 6–8 h per dataset. Future plant research employing micro-CT should strongly consider developing a robust root-tracking approach that is insensitive to artifacts imposed by residual water in the growth medium.

Lastly, the instrument's aperture for accepting samples greatly limited our container size. Future work that employs micro-CT for phenotyping or quantifying belowground phenomena in an undisturbed space must consider the physical size constraints, and perhaps modify their experimental design to ensure that roots remain unimpeded by the boundaries of the container. The containers used for this experiment were sufficiently long (~300 mm), but insufficiently wide (max ~70 mm). Had the container width not been limiting, it is likely that a fewer roots would have been lost during 3D reconstruction.

## Conclusion

In this experiment, we could not conclude with any certainty that intra- and inter-specifically growing seedlings differed in terms of root system architecture and use of 3D space. We showed that, when compared to solitary individuals, inter-specific interactions could have the effect of reducing root production, shifting the depth of root tips, increasing spacing between root tips, and altering the distribution of root system volume over vertical space. Because predictable shifts in rooting depths, lateral root placement, and/or root abundance based on neighbor identity may have far reaching implications in terms of ecosystem function (Hooper et al., 2005), species coexistence (Grime, 1997; Stoll and Prati, 2001; Bruno et al., 2003; Kembel et al., 2008), and plant evolution (Myers et al., 2000), interactions at the community level down to the individual and tissue level must be better understood. The future of this technique is in quantifying both very fine and coarse scale morphological and architectural shifts in root system growth. We demonstrate the ability to quantify 3D parameters and track multiple 3D root systems within a shared volume, which is an important advancement in the field of plant imaging. By coupling CT imaging with algorithms tailored to specific experimental conditions, a wide range of relevant architectural, morphological, and 3D parameters can be analyzed, and the effects of belowground interactions better understood. It is our aim that the marriage of CT with novel algorithms will continue to pave the way toward understanding how plants sense, react, and respond belowground to neighboring plants, and shed light on this highly plastic, ecologically significant, and dynamic process that remains almost entirely unnoticed.

## Author contributions

This work was made possible through intensive, cross-discipline collaboration. Individual contributions are as follows: AP for the experimental design, plant production, data analyses, and lead authorship. JS for 3D metrics, image construction, and major intellectual contributions. JP for intellectual contributions, data processing, and developing novel root-tracking algorithms. TB for intellectual contributions, including experimental design and critical revisions.

### Conflict of interest statement

The authors declare that the research was conducted in the absence of any commercial or financial relationships that could be construed as a potential conflict of interest.

## Supplementary material

Figure S1 **3D reconstruction of all solitary and paired root systems (axes in 0.1 mm increments). (A)** Control spruce (*Picea mariana*) (*n* = 3 containers, 3 individuals). **(B)** Intra-specific spruce (*n* = 3 containers, 6 individuals). **(C)** Inter-specific aspen (*Populus tremuloides*) and spruce root systems (*n* = 3 containers, 3 individuals each species). Aspen is highlighted in orange, spruce is highlighted in blue. **(D)** Intra-specific aspen (*n* = 1 container, 2 individuals). **(E)** Control aspen (*n* = 3 containers, 3 individuals). Note the differences in x- and y-axes.
